# Occupational exposure to fine particulate matter in the reinforced concrete production and its association with respiratory symptoms and lung function

**DOI:** 10.1186/s12889-023-16753-x

**Published:** 2023-09-18

**Authors:** Denis Vinnikov, Anel Abenova, Aizhan Raushanova, Venerando Rapisarda

**Affiliations:** 1 Al-Farabi, Kazakh National University, 71 Al-Farabi Avenue, Almaty, 050040 Kazakhstan; 2 Peoples’ Friendship University of Russia (RUDN University), 6 Miklukho-Maklaya street, Moscow, 117198 Russian Federation; 3 Department of Clinical and Experimental Medicine, Occupational Medicine, University of Catania, Piazza Università, 2, 95131 Catania, Italy

**Keywords:** Occupational, Respirable, Particulate matter, Regression, Spirometry, Reinforced concrete

## Abstract

**Background:**

Reinforced concrete production is widespread, but little is known about the occupational exposure to fine particulate matter (PM) in such workplaces, including from metalworking and concrete processing. Therefore, the aim was to characterize exposure to fine PM in the typical workplaces of the whole production cycle and to quantify the risk of respiratory symptoms and lung function in a cohort of reinforced concrete parts production industry.

**Methods:**

At a reinforced concrete parts producing facility in Almaty, we collected 50 personal PM_2.5_ samples from the main exposure sites and the measured mass concentrations using gravimetric method. Workers also completed questionnaires on a detailed working history, respiratory symptoms (chronic obstructive pulmonary disease (COPD) Assessment Tool (CAT)), followed by spirometry. The association of cumulative dose with CAT score and forced expiratory volume in one second (FEV_1_)/forced vital capacity (FVC) was tested with multiple regression.

**Results:**

The highest PM_2.5_ concentrations were found in the concrete-mixing unit (median 1180 µg/m^3^), followed by metalworking (510 µg/m^3^), armature workshop (375 µg/m^3^) and molding site (245 µg/m^3^), different from the concentrations in the office (29.5 µg/m^3^), Kruskall-Wallis *p* < 0.001. Cumulative PM_2.5_ dose, mg/m^3^-year (beta 0.10 (95% confidence interval (CI) 0.05; 0.15)) was strongly associated with CAT score, whereas production with FEV_1_/FVC (beta -4.96 (-8.31; -1.61)), independent of smoking and chronic bronchitis and sex.

**Conclusions:**

Mixing concrete and metalworks pose the greatest risk for worker’s health in the reinforced concrete production from the inhalational exposure to aerosol, adversely affecting respiratory health.

## Background

Respiratory burden of an occupational disease remains high, and up to 15% of such disease could be prevented if workers were not exposed in the workplace [1]. The burden is most pronounced for occupational chronic obstructive pulmonary disease (COPD), including in the Russian Federation, Kazakhstan and the neighboring countries [2]. Risks are increased by workers’ cigarette smoking, non-optimal use of exposure engineering control measures, and high levels of ambient air pollution [3, 4]. Therefore, the overall burden of chronic respiratory disease, including COPD, is likely underestimated. With the collapse of large industry in the former Soviet countries official employment in the industry in many workers gave way to artisanal production with poor or no exposure control, likely underestimation of true work history and general overlooking of the occupational contribution to COPD and other respiratory diseases. Furthermore, existing misclassification of exposure, likely differential, leads to underestimation of the population involved, the magnitude of doses, their variance and the interaction of such occupational exposure with cigarette and now electronic cigarette smoking.

About one in every four residents in the largest city of Kazakhstan, Almaty, has ever been employed in the industry with some dust, vapor, gas or fume exposure [3]. One of the industries where exposure to dust is likely is reinforced concrete parts production. This industry involves dry cement processing and metalworking, both associated with aerosol production, albeit of differing chemical composition. Exposures in the cement production and processing and welding have been described and discussed widely elsewhere [5–10], whereas workers employed for reinforced concrete parts production may be exposed to both hazards at various stage of such production.

However, descriptive or analytical information about the context and magnitude of exposure to dust in reinforced concrete parts production remains limited. Because exposure assessment data from this industry is lacking, the risk of developing chronic respiratory disease from inhalation exposures in this industry is poorly understood. We found no reports in the international literature presenting quantitative analysis of the concentrations of respirable or fine particulate matter (PM) from the main stages of reinforced concrete parts production, despite wide use of such parts in bridges, houses and industrial infrastructure, and expected large number of people employed in this industry. Furthermore, the analysis of risks in the reinforced concrete parts production would be of great interest specifically for Central Asian countries with the expected current rapid populations growth, which will entail dramatic rise in construction pace resulting in more workers exposed in the newly opened sites. Finally, the individual contribution of occupational exposure and cigarette smoking needs further clarification, but little is known about smoking prevalence and the magnitude of exposure in the workplace in the industry.

To begin to fill these gaps, we performed a cross-sectional study with the aim to characterize exposure to fine PM in the typical workplaces across the production cycle and to quantify the risk of respiratory symptoms and lung function in a cohort of reinforced concrete parts production industry workers.

## Materials and methods

### Study site

The study site was a reinforced concrete products plant located in the industrial zone of Almaty that produces parts for bridges and other products of reinforced concrete. The plant occupies a territory of 7.52 hectares (14,838 m^2^). The plant has 198 permanent staff, but more people may be hired on short-term contracts depending on the workload. Following expert consultation with a plant occupational hygienist, we identified four typical locations of exposure in the production cycle and additionally one more office location as control.

The first location was a concrete-mixing unit, where cement is mixed with other components in rotating tanks, and the PM is generated when loading dry cement and mixing it with water, macadam and other components. Exposures in this location include cement dust, occupational noise and who-body vibration. This process is mostly automatized and does not require human presence in close proximity to the aerosol generation source. Personnel working in this unit enter the location once the mixture is ready, but residual air pollution is present and visible. Compared to other processes, this unit employs the smallest number of workers.

In the second and third locations the primary exposures were to metal aerosols. The second location was the armature shop, where workers knit the structures as future skeletons of reinforced concrete parts from the metal armature. The third location was metalworking workshop, where the structures created in the armature unit are welded. Aerosol in this location is generated mostly from welding, but some metal cutting using plasma cutting machines is also present.

The fourth location was the molding workshop, which employs most of the plant workforce, and in which welded metal skeletons are filled with concrete and molded into the needed shape, then dried and tested. Production occurs both indoors and outdoors, and the outdoor work is prioritized in summer and for large reinforced concrete parts. These workplaces expose workers to cement dust, steam, which is used to consolidate reinforced concrete, and noise. Additionally, final product polishing may generate aerosol.

Finally, the office accommodating accounting, human resources, procurement and other related personnel, was also included in this study as control. There is no vapor, gas, dust or fume exposure in these workplaces, but the office building is located near the main production sites. The plant also accommodates other ancillary departments, units and workshops, such as the canteen, security, boiler plant (steam and hot water are needed to accelerate concrete solidification in the final product) and others.

### Exposure assessment

Exposure data were collected in summer 2023, because otherwise ambient air pollution could have biased the true concentrations given that ambient air pollution levels in Almaty in the heating season are high [4]. In each of the five locations in the plant, we collected ten personal samples using portable PM_2.5_ cyclones and filters. Sampling occurred throughout the full workday, independent on the amount of work and the overall load. Because smoking was not allowed inside production buildings, occupational sources were the only sources of aerosol. Overall, we collected 50 personal samples from workers from five typical locations at the production site. Sampling duration ranged from 6 to 8 h.

Personal samples were obtained from one or two workers from each selected workplace using a portable, battery-powered pump (TM30A-B, TOPSFLO, China) set to provide a flow of 2 l/min for a pre-calibrated cyclone. This portable pump provided a constant air flow through the portable PM_2.5_ cyclone AE2.5-mini (Alaric Electromechanics, USA) to a cassette containing a pre-weighted AФA-BП-20–1 filter (Soyuzhimprom, Russian Federation), and powered from an external battery. This set was placed on a belt of a worker, whereas the edge of the hose was fixed in the breathing zone of a worker. Filters were weighed prior to and after the sample collection using HR-60 (AND, Japan), and the mass concentration of collected PM_2.5_ was calculated as the difference between the filter mass after and prior to sample collection and divided by the overall air volume pumped (in µg/m^3^). For all technical requirements, including the range of acceptable flow rate, recalculation for standard conditions, etc. we followed the actual State Standards (ГOCT) [11, 12].

### Questionnaire and lung function testing

All workers provided informed written consent prior to participation, and the study was approved by the Committee on Bioethics of al-Farabi Kazakh National University. In compliance with the local legislation, all workers, including the office staff, undergo pre-employment and annual medical screening, including spirometry when needed. For the current analysis, we offered a structured validated questionnaire either in Russian or Kazakh to all workers who agreed to participate in this study. Workers completed the questionnaires and performed spirometry in a specially designated office in the administrative building of the plant. This questionnaire aimed to collect a detailed working history ascertaining duration of employment in all previously and currently held positions. In addition to occupational history, we collected information on age, sex, place of residence, smoking history, alcohol use, regular physical activity, and respiratory symptoms. Cigarette smoking status ascertainment yielded stratification into three categories, including current smoking, former smoking and never smoking. Those exercising at least 3 times a week for at least 40 min off work were considered physically active.

Respiratory symptoms were assessed using validated COPD Assessment Test (CAT), which yielded the score from 0 to 40, when the score above 10 was usually indicative of “many symptoms” [13]. The test is useful for chronic obstructive disease management and in cases with already known diagnosis. Dyspnea was measured with widely used mMRC scale, in which the score varies from 0 to 4 [14]. We preferred these two tools in our study for its simplicity and clear score interpretation. We also asked whether a subject had ever had ever been diagnosed with chronic bronchitis, COPD, asthma or allergic rhinitis by a physician.

On the day of examination, workers also asked to refrain from smoking for at least two hours prior to the spirometry test. Three or more reproducible (difference between tests 100 ml or below) maneuvers of vital capacity (VC), followed by the same number of reproducible maneuvers of forced VC (FVC) were completed. We also measured forced expiratory volume in one second (FEV_1_). In subjects with FEV_1_/FVC below 0.7 we had the second set of similar maneuvers following 15 min after inhalation of 300 µg of salbutamol (“Binnopharm”, Russian Federation), and recorded post-bronchodilator readings. We recorded actual values of these volumes, then computed percent predicted values for each subject, except FEV_1_/FVC, using Global Lung Function Initiative (GLI-2012) equations. There were no participants regularly using any bronchodilator, inhaled steroids or other respiratory medication in the study. All tests were performed using MAS 2S office spirometer (Belintelmed, Belarus).

### Statistical analysis

The primary outcome of interest in the association of cumulative exposure to PM_2.5_ in the workplace with current respiratory symptoms and lung function. We were also interested in the quantitative analysis of the magnitude of exposure in range of workplaces within this production.

All variables were evaluated for normality using Shapiro–Wilk test, and the majority of primary variables were non-normally distributed. Hence, we present data as medians with the corresponding interquartile ranges (IQR) or, alternatively, as means with standard deviation (SD). Unless otherwise stated, we utilized and presented the outcomes of non-parametric analytical methods across the manuscript, such as p-values from the Mann–Whitney U-test for continuous two-group data comparison or from χ^2^ test for binary data. In case of normally distributed data analysis, such as for lung function indices, we used t-tests. When more than two groups of non-normally distributed data were compared, we used Kruskall-Wallis test.

Exposure data were summarized as the value in µg/m^3^ for each of 50 days of sampling, including: 10 days from the armature workshop, 10 days from the molding workshop, 10 days from the metalworking workshop, 10 days from the concrete-mixing unit and 10 more days from the office. We then calculated the medians of PM_2.5_ concentrations for each location. We tested whether between-workshop variance exceeded the one within workshops using non-parametric Kruskall-Wallis test and presented the corresponding p-value from the test.

The computed arithmetic mean concentrations for a given workshop were then used to calculate the cumulative dose as a metric of exposure throughout the entire employment for each included worker. Because this specific production needs skilled workers who would unlikely change their occupation throughout their career, we assumed that the current position in the company would be similar to any past employment elsewhere. Therefore, current exposures in the workplace will reflect the picture of exposure a worker have had in the workplace. Hence, exposure metric used and analyzed in this study is the function of PM_2.5_ concentration in the current position and the overall work duration (cumulative PM_2.5_ dose, mg/m^3^-year).

The final stage in the analysis was testing the association of cumulative exposure with the outcomes of interest, including CAT score as a surrogate of respiratory symptoms and FEV_1_/FVC using linear regression models. First, we confirmed the effect of selected variables in the univariate analysis, including age, female sex, cumulative dose, chronic bronchitis and current cigarette smoking with CAT as an outcome. For FEV_1_/FVC, the range of such predictors was narrower and only comprised cumulative dose and production group. We then included them in the adjusted models adjusted for the total of three, except age (in case of CAT) and two (in case of FEV_1_/FVC) variables. We report beta coefficients for significant (*p* < 0.05) predictors from the adjusted for confounders models with the corresponding 95% confidence intervals (CI). All tests were completed in NCSS 2021 (Utah, USA), and *p*-values below 0.05 were considered significant.

## Results

### Overall workforce portrait

A total of 162 agreed to participate in this study out of 198 permanent staff (response rate 82%). Most included workers (Table 1) at the time of this study were in their 40 s with the median of 19 (IQR 10;30) years in service overall with significant difference between occupational groups. Age is highly correlated with the total years of work (*r* = 0.91), however the correlation of years in work at the plant had a weaker correlation with age (*r* = 0.43) and overall years in service (*r* = 0.44). Most workers at the plant were either current or ex-smokers (58%), used alcohol (61%) and were not active physically in their leisure time (79%). Workers from the different locations in the plant, differed in the years in service at the plant in their current position; the shortest employment duration was in the office, followed by the molding workshop. Groups did not differ in alcohol use and physical activity between each other.

**Table 1:** Demographic and occupational profile of included staff

	Overall	Armature workshop	Molding workshop	Metalworking workshop	Concrete-mixing unit	Office
N (%)	162 (100)	39 (24)	39 (24)	46 (28)	8 (5)	30 (19)
Males, N (%)*	115 (71)	28 (72)	22 (56)	45 (98)	8 (100)	12 (40)
Age, years*	45 (34;51)	44.2 ± 12.1	38.9 ± 9.6	46 (36;55)	38.5 (34.5;46.8)	44 (36;51)
Years in service total*	19 (10;30)	19 (10;30)	11 (8;22)	29 (14.5;36.8)	18 (11;29.5)	19.5 (13.8;26.8)
Years in service at the plant*	4 (1;8)	8 (4;15)	2 (0.8;6)	4.5 (1.5;10)	5 (3.3;9.5)	1 (1;6)
Cigarette smoking, N (%)*						
Never	66 (42)	16 (41)	20 (51)	9 (20)	1 (12)	20 (67)
Ex-smoking	37 (22)	9 (23)	7 (18)	19 (41)	1 (13)	1 (3)
Current smoking	59 (36)	14 (36)	12 (31)	18 (39)	6 (75)	9 (30)
Never-alcohol use, N (%)	63 (39)	20 (51)	15 (38)	13 (28)	2 (25)	13 (43)
Regular physical activity, N (%)	34 (21)	8 (21)	5 (13)	11 (24)	1 (12)	9 (30)

Continuous data shown either as medians with the corresponding interquartile range in case of non-normal distribution or as means ± standard deviation in case of normal distribution; *—*p* < 0.05 using either Kruskall-Wallis test or χ^2^ test

### Exposure data

We collected a total of 50 PM_2.5_ personal samples in four production workshops and one control non-production location. Sampling was completed in August 2023. We found low variability in the office, where daily concentrations ranged from 17 to 45 (median 29.5) µg/m^3^ (Table 2). Workers’ exposure was the highest in the concrete-mixing unit with greater variability, and the concentrations ranged from 980 to 1670 µg/m^3^ (Table 2). The second location with top exposure to aerosol was a metalworking unit with most workers involved in welding and metal cutting (median PM_2.5_ concentration was 510 µg/m^3^).

**Table 2:** Daily concentrations of PM_2.5_ in the studied locations at the reinforced concrete production site with the summary median concentrations in µg/m^3^

	Armature workshop	Molding workshop	Metalworking workshop	Concrete-mixing unit	Office
Day 1	180	150	480	1080	18
Day 2	360	260	540	1320	38
Day 3	400	180	400	1540	41
Day 4	370	380	280	990	28
Day 5	290	250	970	1020	31
Day 6	510	150	320	1670	45
Day 7	580	240	740	980	21
Day 8	470	300	610	1120	28
Day 9	380	200	680	1520	33
Day 10	330	260	450	1240	17
Median of all 1-min data points*	375	245	510	1180	29.5

^*^—*p* < 0.001 in Kruskall-Wallis test; concentrations are in µg/m^3^

**Fig. 1:**
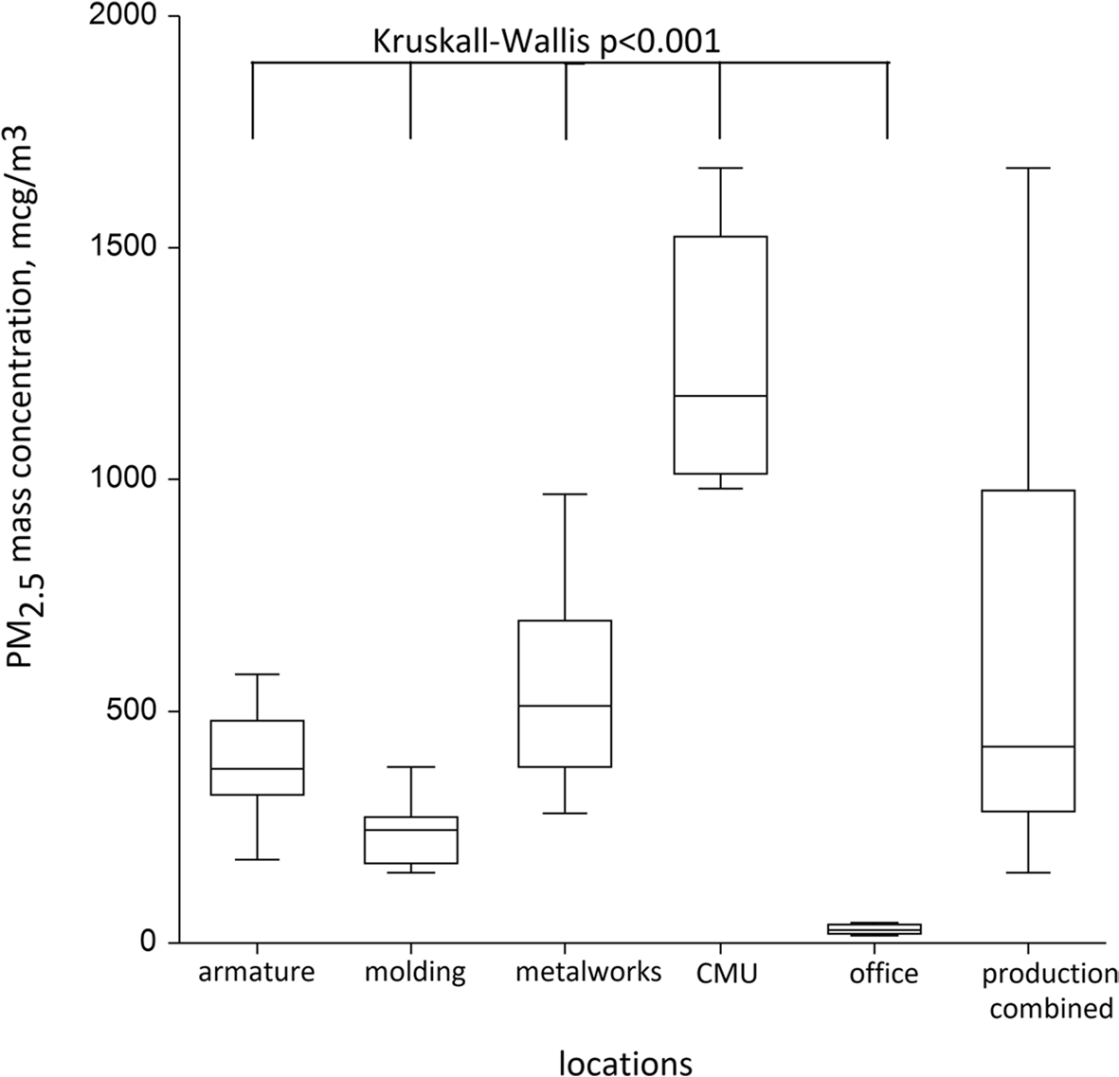
Distribution of PM_2.5_ mass concentrations (µg/m^3^) at study locations

The armature and molding workshops were sites with the lowest PM_2.5_ exposure among the production workshops, with median PM_2.5_ concentrations 375 and 245 µg/m^3^, accordingly. Compared to production sites, the overall exposure to aerosol was the least in the office, where the median concentration was almost 40 times lower compared to the concrete-mixing unit. When sampling locations were compared to each other, we found a highly significant difference between the median concentrations (Kruskall-Wallis *p* < 0.001). Figure 1 illustrates dramatic difference between included locations, showing that highly significant differences were found not only when comparing all five included locations, but when only production sites were compared with each other.

### Respiratory health and dose – respiratory response association

CAT score ranged from 0 to 18, and the score above 10 was found in only 5 workers, all from production staff (Table 3). Dyspnea score was very low in the whole group, where score 0 corresponded to 95^th^ percentile, similar in both production and non-production employees. Both low CAT and mMRC were in general indicative of very low prevalence of respiratory symptoms. Among the four self-reported respiratory diagnoses, chronic bronchitis was the most prevalent, and almost 11% of workers had this diagnosis before. Allergic rhinitis was ever diagnosed in almost 10% of employees. COPD was mentioned by one worker only. No bronchial asthma was ever diagnosed in the studied group. Table 3 also summarizes lung function data, demonstrating that the group in general had indices far beyond the threshold for abnormality, including below 80% for FEV_1_ and FVC and 70% for FEV_1_/FVC Interestingly, we found significantly greater FVC% predicted in the production workers, which obviously resulted in significantly lower FEV_1_/FVC in this group. Overall, we confirmed COPD with spirometry in 11 workers, none of which had this diagnosis before and none was on any treatment for COPD. Cumulative PM_2.5_ dose ranged from 150 to 62400 µg/^3^*year^−1^ (median 6027, IQR 1641 to 13949) with significant difference between the studied groups, with the overall pattern of difference similar to the difference in the exposure data (Fig. 1).

**Table 3:** Respiratory symptoms along with respiratory ever-diagnoses and lung function parameters in the studied sample

	Overall	Production	Office	p
N (%)	162 (100)	132 (81.5)	30 (18.5)	-
CAT score, range 0–40	2 (1–4)	2 (1–4)	2.5 (1–4)	NS
Chronic bronchitis, N (%)	17 (10.5)	14 (10.6)	3 (10.0)	NS
COPD, N (%)	1 (0.6)	1 (0.8)	0 (0)	NS
Asthma, N (%)	0 (0)	0 (0)	0 (0)	NS
Allergic rhinitis, N (%)	15 (9.3)	13 (9.8)	2 (6.7)	NS
FVC, % predicted	89.2 ± 13.1	90.8 ± 12.2	81.8 ± 14.8	< 0.01
FEV_1_, % predicted	89.9 ± 14.5	90.7 ± 14.2	86.0 ± 15.6	NS
FEV_1_/FVC, %	83 (78; 87)	82 (77.3; 86)	85.5 (82; 93)	< 0.001

*COPD* chronic obstructive pulmonary disease, *CAT* COPD Assessment Test, *FEV*_*1*_ forced expiratory volume in one second, *FVC* forced vital capacity; continuous data shown either as medians with the corresponding interquartile range in case of non-normal distribution or as means ± standard deviation in case of normal distribution, *NS* non-significant; *P*-values were either from t-test or Mann–Whitney test depending on distribution for continuous variables or, alternatively, χ2 test for binary data

When CAT score was analyzed as an outcome in simple regression models, age, sex, current cigarette smoking, cumulative PM_2.5_ dose and ever-chronic bronchitis, but not occupational groups, were positively associated with CAT score in the univariate analyses (Table 4). Significant predictors from the univariate analyses were then included in the adjusted models, except age. Age was not included in the CAT model to suppress the effect of collinearity, because cumulative dose was also a function of years worked, highly correlated with age. In such adjusted models, cumulative PM_2.5_ dose, sex, current smoking and chronic bronchitis had a significant association with CAT score (Table 4), and this combination explained 30% of all CAT variability. In current smokers, CAT score was significantly greater, whereas FEV_1_/FVC was lower, independent of production group, cumulative aerosol dose and chronic bronchitis. As opposed to respiratory symptoms, none of four included respiratory diagnoses were associated with FEV_1_/FVC in the univariate comparisons, as weren’t the age, female sex or even cumulative PM_2.5_ dose. However, production group demonstrated strong association with reduced FEV_1_/FVC independent of cumulative dose and smoking (Table 4). Between-group comparisons did not confirm the association of an occupational group with CAT score; however, similar Kruskall-Wallis was highly significant for FEV_1_/FVC as a dependent variable. Post hoc test confirmed significantly lower FEV_1_/FVC in a metalworking group compared to the office group.

**Table 4:** Beta coefficients from crude and adjusted regression models with their corresponding 95% confidence intervals

Factor	CAT score		FEV_1_/FVC	
	Crude	Adjusted	Crude	Adjusted
Age	0.08 (0.04;0.13)	Not included	-0.10 (-0.22;0.02)	Not included
Female sex	1.17 (0.07;2.28)	1.62 (0.62;2.64)	1.46 (-1.46;4.39)	Not included
Cumulative PM_2.5_ dose, mg/m^3^-year	0.11 (0.05;0.16)	0.10 (0.05; 0.15)	-0.13 (-0.28;1.01)	Not included
Current smoking	1.68 (0.66;2.70)	1.876 (0.85; 2.67)	-0.72 (-3.48;2.04)	-0.46 (-3.17;2.24)
Chronic bronchitis	4.02 (2.48;5.55)	3.43 (1.99; 4.87)	-3.44 (-7.75;0.87)	Not included
Production work (reference – office)	0.12 (-1.19;1.43)	Not included	-5.00 (-8.33;-1.66)	-4.96 (-8.31; -1.61)
Armature workshop (reference – other groups)	0.22 (-0.96;1.41)	Not included	-1.87 (-4.97;1.23)	Not included
Molding workshop (reference – other groups)	-0.45 (-1.63;0.74)	Not included	-1.53 (-4.64;1.57)	Not included
Metalworking workshop (reference – other groups)	0.24 (-0.88;1.37)	Not included	-1.99 (-4.92;0.95)	Not included
Concrete-mixing unit (reference – other groups)	0.20 (-2.14;2.55)	Not included	5.81 (-0.27;11.88)	Not included
Office (reference – other groups)	-0.12 (-1.43;1.19)	Not included	5.00 (1.66;8.33)	Not included

*FEV*_*1*_ forced expiratory volume in one second, *FVC* forced vital capacity, *CAT* COPD Assessment Test, *PM* particulate matter. CAT model is adjusted for cumulative PM_2.5_ dose, current smoking and chronic bronchitis; FEV_1_/FVC model adjusted for current smoking and production work

## Discussion

This is a presentation of a cross-sectional study at a reinforced concrete production plant in Almaty, where we analyzed exposure to PM_2.5_ in the workplaces, which represented the main sources of aerosol generation. These processes included concrete mixing, welding, metalworks, armature knitting and product molding. We showed that concrete mixing and metalworking were the processes generating the highest concentrations of PM_2.5_. Additionally, we also demonstrated that such cumulative exposure to PM_2.5_ negatively affected respiratory symptoms in a linear manner, whereas work in production increased the risk of bronchial obstruction independent of smoking.

Aerosol generated in the reinforced concrete parts production may have different chemical composition representing various processes, predominantly metalworking and concrete mixing. We found that PM_2.5_ concentrations during welding and other metalworks in the current location were quite close to the ones from similar metalworking processes elsewhere [8, 15]. Such aerosol will expose workers to metals and their oxides, which are expected to induce inflammatory response and increase the risk of typical health outcomes, usually found in welders [7, 16–19]. As opposed to typical exposure in metalworking and welding, the concentrations during concrete mixing and final reinforced concrete product molding remained unknown.

From a very few published studies, exposure to dust in cement production is considered very high [5], and the associated health effects are first expected to be respiratory, such as accelerated lung function decline [5, 6, 9, 20]. In a meta-analysis they found a mean difference of -0.7 (95% CI -0.92 to -0.47) litres for FEV_1_, whereas another systematic review demonstrated that FEV_1_/FVC was 1–6% lower in those exposed compared to controls in the cross-sectional studies, and yearly decline was 0.8–1.5% in the cohort studies [20]. Furthermore, the concentrations of respirable dust during cutting reinforced concrete, which is also expected to emit crystalline silica, have been presented and were as high as the geometric mean of 14.4 mg/m^3^, almost tenfold lower when wet cutting is used [10]. Health effects of this specific aerosol from the reinforced concrete demolition were also poorly described, but one of those effects characterized in the literature was the TNF-α release from exposure to fine PM in the animal models [21]. Exposure to dust, including cement dust in the reinforced concrete production, however, remained poorly studied and analyzed in the scientific literature.

Our data showed that reinforced concrete production may expose workers to much lower concentrations of fine PM compared to cement production or cutting reinforced concrete; however, this exposure is non-uniform and the risk may be much greater in the workplaces where dry cement is used, such as at the stage of cement mixing to form concrete. Given that among two workplaces with the highest level of exposure, including concrete mixing and metalworking, only few worked in the former, the contribution of metalworking and welding in the overall workers’ exposure will prevail. Therefore, reinforced concrete parts production should be considered the one with predominantly metalworking and welding exposures as the major hazards of inhalational route. Because health effects of welding, both chronic disease and malignancies, have been widely described in the literature [7, 16–19, 22–24], engineering, organizational and personal exposure control measures should be prioritized for the metalworking component of reinforced concrete part production. Respiratory personal protective equipment (PPE) should be widely used, given that the individual metal absorption in metalworkers and welders may be significant [25] and respirators have been shown efficient with regard to welding fumes [26]. As opposed to production sites, the concentrations in the office in general were close to the overall baseline ambient air pollution levels in Almaty in the non-heating season.

We believe that the inclusion of all technological processes of a complete reinforced concrete products production cycle at one location in the current analysis is a strength of our presentation. Sufficient number of PM_2.5_ samples, representative of all major production processes, is also a value of our research project. Our study, has, however, a number of limitations. Firstly, although we included all personnel willing to participate and the response rate was high, the overall study sample size may be lower when compared to population-based and industry-based studies. Secondly, cross-sectional design did not allow to verify the incidence of respiratory effects of interest, such as COPD, because patients may had entered the job already with symptoms, such as atopic asthma from the childhood. Thirdly, women were underrepresented in this occupational cohort, but this effect was prevalent in most industrial places with large physical demands. Furthermore, we could not perform sampling at one more location of potential exposure, such as boiler unit, but the loss of data value is small because only four workers were regularly employed there.

In addition, some improvements in equipment and production process may decrease exposure with time; however, no measurements, including PM mass concentrations in the workplace, were available from the previous employment of participating workers, and we could not go any further in calculating their estimated lifetime exposure. In addition, smoking intensity and years of smoking were collected from the current smokers only, thus, underestimating the contribution of smoking history in former smokers if pack-years were used as a confounder in regression models. Hence, we believe that smoking was not significantly associated with lung function in our sample because of healthy worker survival effect, often found in the occupational settings [27], and because accumulated smoking history could not be thus considered in former smokers. Finally, cross-sectional design of this study did not allow to verify temporality and observe workers with elapsing time.

Our study has clear implications for occupational health from the perspective of public health preventive measures. Firstly, wider use of engineering control measures to reduce exposure, such as automated welding machines, ventilation and better welding technology, may further reduce the risk, and future studies should document such effect. Secondly, in addition to wider use of PPE in the reinforced concrete parts production, regular surveillance of workers, more frequent in those employed at the concrete-mixing unit, welding and metalworking, should be reinforced and controlled by the company management. Annual lung function decline is important as an early obstructive ventilation defect detection in the occupational settings [28]. In Kazakhstan [3], as elsewhere in the neighboring countries [2], the occupational burden of respiratory disease remains high, most notable for COPD, when lifetime occupational history as a welder could increase the risk fivefold (adjusted relative risk 4.98 (95% CI 1.87–13.29) [3]. Smoking cessation in these workers’ groups should be prioritized, possibly with a clear mechanism of reward incentives, because further effect amplification, when smoking adds to inhalational occupational exposures, has been demonstrated in a great number of studies with high level of evidence. Future studies should not be limited to the respiratory outcomes of the reinforced concrete parts production, but should also encompass cardiovascular effects of the fine PM of this specific chemical composition.

## Conclusions

This first study to characterize main processes and the associated occupational exposure to PM_2.5_ at a reinforced concrete parts production has identified locations with high exposure, including concrete-mixing unit and a metalworking workshop. These exposures were associated with more respiratory symptoms and reduction in FEV_1_/FVC, independent of smoking and past respiratory diagnoses. Smoking cessation, wider use of PPE and other exposure control measures should be widely used in the reinforced concrete parts production aiming to preserve workers’ health.

## Data Availability

The datasets used and/or analysed during the current study are available from the corresponding author on reasonable request.

## Abbreviations

CAT: COPD Assessment Test
CI: Confidence interval
COPD: Chronic obstructive pulmonary disease
FEV_1_: Forced expiratory volume in one second
FVC: Forced vital capacity
GLI: Global Lung Function Initiative
GM: Geometric mean
IQR: Interquartile range
PM: Particulate matter
PPE: Personal protective equipment
SD: Standard deviation
